# Our Delay

**Published:** 1853-03

**Authors:** 


### OUR DELAY.

It cannot have escaped the remark of our readers, that our issues have been for some time past growing progressively more tardy, until at length we have fallen more than a month behind our regular day of publication. We assure them the fault is not with us; and we also beg them to believe that this circumstance, however unpromising, is not an indication that our work is about to be discontinued. The delay has been occasioned by the press of work at the office where the Journal is printed, and we have the pledge of the publishers that in future they will observe greater punctuality.

